# Harmonic trophic potentials and memory effects in ecological dynamics in resource-limited ecosystems through the Generalized Lotka–Volterra model with biomass constraints

**DOI:** 10.1007/s12064-026-00459-w

**Published:** 2026-03-11

**Authors:** Josenilson Adnei Oliveira Marinho, Herson Oliveira da Rocha, Fernando Pereira Paulucio Reis

**Affiliations:** 1 Environmental, Geosciences, and Engineering Study Group (GEMAGE), Federal Rural University of Amazonia (UFRA), Avenue Duane Silva Sousa, S/N, Road PA 275, Parauapebas, Pará 68515-000 Brazil; 2 Postgraduate Program in Physics, Federal University of Southern and Southeastern Pará (UNIFESSPA), Avenue Paulo Fonteles, S/N, Marabá, Pará 68.500-000 Brazil; 3 Polytechnic Institute (IPOLI), Federal University of Rio de Janeiro (UFRJ), Avenue Aluizio da Silva Gomes, S/N, Macae, Rio de Janeiro 27930-560 Brazil

**Keywords:** Generalized Lotka–Volterra model, Trophic dynamics, Biomass constraints, Harmonic functions, Ecological stability, Thermodynamic equilibrium

## Abstract

This study investigates the thermodynamic and mathematical foundations of trophic dynamics in ecological systems, focusing on the Generalized Lotka–Volterra (GLV) model to analyze prey/predator interactions under resource constraints. We introduce a framework where species’ contributions are governed by mean biomass and relative trophic strength, subject to physiological and ecological bounds. The total biomass function and abiotic reservoir are defined to quantify the balance between biotic and abiotic resources, with the functional capture of net energetic flux. Key results demonstrate that dynamic equilibrium is achievable only in trivial cases (extinction) when measures of historical interactions are absolutely continuous. In contrast, memory-free models, where species interactions are instantaneous, permit nontrivial equilibria under harmonic conditions, for the effective trophic potential harmonicity implies local equilibrium, ensuring smooth integration of species into the trophic network without abrupt disruptions. A two-species system (in a bidimensional case) illustrates phase space dynamics: Stability analysis highlights the sensitivity of ecosystems to competitive dynamics and resource influx, with structured ecological inputs that promote equilibrium. The study bridges theoretical ecology and dynamical systems, offering insight into the stability of trophic networks under varying constraints and setting an effective machinery that establishes a clear connection between classical GLV models and mass balance treatments in ecosystems.

## Introduction

The classical problem of two species competing, namely the pair of coupled differential equations,

1.1 $$\begin{aligned} {\left\{ \begin{array}{ll} \displaystyle {\frac{dN}{dt}= N\left[ \lambda - a N\right] -bNP} \\ \frac{dP}{dt}=P\left[ \mu -kP\right] +cNP \end{array}\right. } \end{aligned}$$

usually called Lotka–Volterra equations, establishes the basic variables describing the dynamics of a predator P feeding on a prey N. The results arising from the mathematical treatment of such a dynamical system yield a rich and interesting case for discussion and application, bringing to attention the behavior of species in a delicate game of ecological equilibrium. In particular, prey/predator systems have served as a fundamental testing ground for both theoretical and empirical models that explore the emergence of stability, oscillatory behavior, and collapse within ecological communities. A straightfoward generalization for the LV equations, the Generalized Lotka–Volterra (GLV) model, which captures the interdependence of species through nonlinear differential equations governed by pairwise interaction coefficients, describes a web composed by n species (Turney 2019; Raman 2021), according to Eq. (1.2):

1.2 $$\begin{aligned} \frac{dN_i}{dt} = N_i \left( r_i + \sum _{j=1}^n a_{ij} N_j \right) \end{aligned}$$

However, the rising problem of making predictions in ecology and environmental dynamics in the present days of climate changes is a compelling challenge that demands a realistic approach, accounting for the natural resources available for the ecological systems, such as energy and matter. Recent efforts have encompassed energy conservation and information content, motivated by the recognition that trophic activity must be sought as a thermodynamical system, both in fishery theory (Lucey et al. 2020) and also for new mathematical formulations in applied ecosystems (Thorson et al. 2025). In this context, ecosystem dynamics are shaped by a balance between internal resource consumption and external energy inflow, and the viability of species is regulated by both trophic structure and environmental pressure (Caporali 2021; Jordan 2021; Furnell 2022).

Then, despite the present approach handling the machinery provided by the classical GLV framework, a seminal contribution is given by the mathematical formulation of mass and energy current conservation by bounding to the model the equation ruling the flow of matter within and in or out the system for the inner degrees of freedom pertaining to the dynamical system of multiple competitors. Thus, one can argue that the internal dynamics behave according to the flow of energy/matter in or out and introduce modifications in the neighborhood in turn. The coupling with the new terms goes beyond this driven dynamics responding to the available resources: it also includes external income of energy, hence rendering the system open. This means we can deal in a more realistic way with solar (photosynthetic for instance) source as a major player in the game, just as many Mass Balance models usually do. In this sense, this approach covers a program aiming to build a rigorous bridge between GLV models and Mass Balance models much in the path of Walters et al. (1997).

In order to deal with the effective size of the species, we define a new trophic functional $\tau _i$ that quantifies the cumulative ecological impact of a species over time, taking into account its mean biomass $\bar{m}_i$ and relative trophic strength $k_i$, so it assumes, under certain conditions, the form $\tau _i =\bar{m}_i N_i k_i$. As long as the product between the mean mass and the number of individuals may be seen as the species mass, $M_i =N_i\bar{m}_i$ (so one gets the form $\tau _i =M_i k_i$) we may conclude that $\tau _i$ is a momentum (p-number) if we can interpret $k_i$ as a dynamical variable in the phase space equivalent to a velocity (time derivative of a q-number generalized coordinate). And the set of p-numbers $\tau _i$, for our purpose, may represent a system of n-generalized coordinates describing a gas of n particles moving in a 1-D box.

The mechanical analogy (where $\tau _{i}$ acts as a moment and *Y* as an external force) serves primarily to structure the balance of forces in the trophic network mathematically. However, its paramount ecological significance lies in providing a unified formulation for energy flow, allowing the derivation of the $Y=\frac{d}{dt}[M - R]$ continuity equation. Thus, the mechanical formalism is not an end in itself, but a means to translate ecological interactions into quantitative relationships between biomass, abiotic reserve, and external energy influx, central elements for a thermodynamically grounded analysis.

Then this highlights the mechanical content of the formulation, since analytically our Newton’s second law of motion is set up as the external influence (the force *Y*) producing the dynamical variation of momentum:

1.3 $$\begin{aligned} Y := \sum _i \frac{d\tau _i}{dt}. \end{aligned}$$

This statement, from the point of view of causality, is fundamental as long as the external force will cause displacement in the internal configuration of the system, i.e., the distribution of inner momenta $\tau _i$. Epistemologically, we know, on the other hand, that the origin of forces must be known so that one can determine the way the system will alter its configuration. In practice, however, it may turn out to be quite hard to determine the external forces acting over the ecosystem, so we must handle the thermodynamic variables available.

In what concerns the mathematical formulation, these tools need to be equipped with a constraint brought by the bounded quantity of physical resources, so we claim the conservation of the total amount of organic matter given by the sum of all biomass stored in the species $\sum _i \tau _i$, along with the reservoir *R* of organic matter of the ecosystem, and this total quantity sums up to a conserved quantity *M*:$\sum _i \tau _i + R = M$. Straightforwardly, the continuity equation arisen tells us that the increment of biomass will be included in the species or will be converted into more biomass stocked into the reservoir:

1.4 $$\begin{aligned} \frac{dM}{dt}=\sum _i \frac{d\tau _i}{dt} + \frac{dR}{dt}. \end{aligned}$$

Thus, the only way to increase or decrease the total amount of biomass in the system takes place by a equivalent Newton’s second law of dynamics, by varying the total *M* (external force) while converting inorganic matter (from outside the system) into organic one (using external energy) or either dismissing organic matter turned into inorganic one (with release of energy and matter to the environment). We can construct the external force from the continuity equation;

1.5 $$\begin{aligned} Y= \frac{d}{dt}[ M - R], \end{aligned}$$

which encodes the net rate of energetic exchange between the abiotic neighborhood and the biological subsystem. The sign of *Y* provides crucial insight into whether the ecosystem is experiencing accumulation, depletion, or equilibrium in terms of energy and matter. We investigate the regularity, solvability, and interpretive consequences of *Y* under various modeling assumptions.

In addition, for the sake of generality, we have a completely arbitrary set of equations with several applications. The web $\tau _1, \tau _2, \ldots, \tau _n$ indeed does not depend on any special trophic relation: it does not matter who preys whom, but rather what is each relative trophic strength. In this game, one has n competitors fighting for resources—they can be n countries, or n football players, or even n trade makers in a closed market. In this scenario, the abstract construction of each species takes into account only the quantity of resources consumed by each specific species *i*, and this quantity determines its relative size.

Although the GLV model is traditionally studied through dynamical systems theory, our approach explicitly incorporates the mass and energy balance between the biotic and abiotic components of the ecosystem, represented by the functions *M*(*t*) and *R*(*t*). This innovation allows us to quantify the net energy flow (*Y*) between the abiotic reservoir and the trophic network, something that standard GLV models do not directly capture. By imposing physiological constraints (via the $C_{i}$ limit) and considering regimes with and without historical memory, this study provides a more realistic representation of resource-limited ecosystems, where total biomass availability and energy storage/release dynamics are critical factors for stability. Therefore, the present formulation not only analyzes population dynamics but also grounds them in thermodynamic and mass conservation principles, offering a bridge between classical interaction modeling and ecosystem energy budgets.

Another interesting feature we will explore in this work is the historical relevance of each p-number $\tau _i$, which resembles the recognition of the prevalence of each species’ status on the web. The analysis distinguishes between memory-dependent ecosystems, where species’ trophic functionals incorporate historical interactions via weighted integrals, and memory-free regimes, where immediate state variables determine interaction strength. In particular, we demonstrate that thermodynamic equilibrium in memory-dependent models is only attained in the trivial extinction case, whereas memory-free systems permit non-trivial equilibrium, provided suitable conditions are met on the trophic potential.

This study proposes a thermodynamic–mathematical framework to analyze trophic dynamics in resource-limited ecosystems, integrating the Generalized Lotka–Volterra (GLV) model with an explicit biomass and energy balance between biotic and abiotic components. Our main objective is to formalize and ecologically interpret the functionality of energy flow, which quantifies the net energy exchange between the abiotic reservoir and the trophic network. We seek to demonstrate how different historical memory regimes (models with absolutely continuous measures versus models without memory) influence the possibility of non-trivial equilibrium.

The structure of this manuscript is organized as follows: Sect. 1 introduces the main components of the GLV models. At the same time, the regularity criteria and the trophic functional $\tau _{i}$ are defined in Sect. 2. In Sect. 3, the reserve function *R* and the total biomass function *M* are introduced, the flow functional *Y* is derived, and Theorem 3.1 on equilibrium in models with memory is presented. The model without memory and the trophic potential’s harmonicity property are covered in Sect. 4, which ends with Theorem 4.1. In Sect. 5, phase portraiture and stability are examined in the two-dimensional situation (prey/predator) using the generic framework. The energy and mass flow through scenarios for $Y>0$, $Y<0$, and $Y=0$ are covered in Sect. 6. Section 7 concludes with talks and outlooks for the future study.

## The trophic functional

Consider the Generalized Lotka–Volterra (GLV) system

2.1 $$\begin{aligned} {\left\{ \begin{array}{ll} \displaystyle {\frac{dN_i}{dt} = N_i \left( r_i + \sum _{j=1}^n a_{ij} N_j \right) , \quad i = 1, \dots , n,} \\ N(0) = N_0 \in \mathbb {R}^n_{> 0}, \end{array}\right. } \end{aligned}$$

where $\mathbb {R}^n_{> 0} = \{ x \in \mathbb {R}^n : x_i> 0 \}$. Let $I_{N_0}$ be the maximal interval on which the unique solution $N(t; N_0)$ to (2.1), passing through $N_0$ at time $t_0$. Define

$$\begin{aligned} \mathcal {T}_{N_0} : \{N(t,N_0) : t\in I_{N_0}\} \end{aligned}$$

the trajectory through $N_0$ and

$$\begin{aligned} \mathcal {S} := \bigcup _{N_0 \in \mathbb {R}^n_+} \left( I_{N_0} \times \mathcal {T}_{N_0} \} \right) \subset \mathbb {R} \times \mathbb {R}^n_+. \end{aligned}$$

Let $\bar{m}_i, k_i : \mathcal {S} \rightarrow \mathbb {R}_+$ be smooth functions representing, respectively, the mean biomass and the relative trophic strength of species $i$. We assume that, for every $(t, N(t; N_0)) \in \mathcal {S}$, the product satisfies

2.2 $$\begin{aligned} \bar{m}_i(t, N(t; N_0)) \cdot k_i(t, N(t; N_0)) \le C_i(N_0), \end{aligned}$$

for some constant $C_i(N_0)> 0$ depending on the initial condition $N_0$.

### Remark 2.1

The upper bound (2.2) reflects physiological and ecological constraints that limit the functional biomass and relative trophic influence of species $i$. Imposing such bounds is consistent with empirical observations and theoretical models (Li et al. 2021; Whittaker and Marks 1975; Society 2023).

### Proposition 2.1

Let $\{\mu _t\}_{t \in (0,+\infty )}$ be a family of finite positive Borel measures supported on the interval $I_t := [0, t] \subset \mathbb {R}_{+}$ for each $t> 0$. Then, for each $1 \le i \le n$, the functional

$$\begin{aligned} & \tau _i(t, N(t; N_0)) := \frac{1}{t} \int _{I_t} \bar{m}_i(s, N(s; N_0))\,\\ & k_i(s, N(s; N_0))\, N_i(s; N_0)\, d\mu _t(s) \end{aligned}$$

is well defined for all $N \in \mathcal {S}$.

### Proof

Let $i \in \{1, \dots , n\}$ be fixed. By assumption (2.2) (see also Remark 2.1), there exists a constant $C_i(N_0)> 0$ such that

$$\begin{aligned}&\bar{m}_i(s, N(s; N_0))\, k_i(s, N(s; N_0)) \\&\le C_i(N_0), \quad \text {for all } s \in [0, t]. \end{aligned}$$

It follows that

$$\begin{aligned}&\int _{ I_t } \bar{m}_i(s, N(s; N_0))\, k_i(s, N(s; N_0))\, \\&N_i(s; N_0)\, d\mu _t \le C_i(N_0) \int _{ I_t } N_i(s; N_0)\, d\mu _t. \end{aligned}$$

Since $N_i(\cdot ; N_0)$ is continuous on $[0,t]$, and $\mu _t$ is a finite Borel measure with compact support in $[0,t]$, it follows that the function $s \mapsto N_i(s; N_0)$ is Borel measurable on the support of $\mu _t$, and hence integrable. Therefore, since $N\in \mathcal {S}$ then $\tau _i(t,N(t; N_0))$ is well defined for all $\displaystyle t \in [0,+\infty )$. $\square$

Consider the subset

$$\begin{aligned} \mathcal {S}' = \left\{ I_{N_0} \times \mathcal {T}_{N_0}\in \mathcal {S} \; : \; I_{N_0}= [0, +\infty ) \right\} . \end{aligned}$$

From an ecological perspective, it is natural to assume that the set $\mathcal {S}'$ is nonempty. Indeed, even if certain populations eventually go extinct, the corresponding solution should remain well defined for all finite times, as long as the species existed at some point in the past. In this context, extinction corresponds to the solution approaching or reaching a coordinate hyperplane, without implying blow-up or ill-posedness of the system.

From a mathematical viewpoint, there are classical conditions that guarantee the existence of global solutions. For instance, suppose that for some initial condition $N_0 \in \mathbb {R}^n_{>0}$, there exists a compact subset $C \subset \mathbb {R}^n_{>0}$ such that the solution $N(t)$ of the GLV system (2.1) with $N_0\in C$ satisfies $N(t) \in C \quad \text {for all } t \in [0, \beta ),$ for some $\beta < +\infty$. Under these conditions (see (Hirsch et al. 2013, p. 399)), the solution can be extended to all $t \in [0,+\infty )$ and is therefore globally defined. See also (Thieme 2003, p. 424). From now on, we suppose that $\mathcal {S}'$ is nonempty.

## Flow of energy and matter

We now define the *total biomass function* $M : [0, +\infty ) \rightarrow \mathbb {R}_{>0}$ as a prescribed function of class $\mathcal {C}^1\left( [0, +\infty )\right)$, which assigns to each time $t$ the total amount of biomass available in the ecosystem. This quantity includes both the abiotic mass and the trophically allocated biomass distributed among all species. Hence, we impose $M(t)\ge \sum _{i=1}^n\tau _i(t,N(t,N_0))$ for all $t,N(t,N_0)\in \mathcal {S}'$. Accordingly, we define the abiotic reservoir function $R : \mathcal {S}' \rightarrow \mathbb {R}_{\ge 0}$ by

3.1 $$\begin{aligned} R(t, N(t; N_0)) := M(t) - \sum _{i=1}^n \tau _i(t, N(t; N_0)), \end{aligned}$$

where $R$ represents the fraction of total biomass that remains unallocated to biological activity, interpreted as the abiotic reserve. Note that although *R* depends on the population trajectory *N*(*t*) via the functional terms $\tau _i$, the total biomass *M*(*t*) does not. This distinction reflects an important ecological principle: in many realistic scenarios, resource influx (e.g., photosynthetic production, external nutrient loading) is governed by abiotic processes and environmental forcing that are independent of the internal state of the system. Furthermore, for the trivial solution $N(t) \equiv 0$, we have $R(t, {\textbf {0}}) = M(t)$, which represents the extinction of all species and implies that the abiotic biomass reserve coincides with the total biomass of the system.

### Remark 3.1

Let $F(t, \textbf{x})$ be a smooth function depending on the variable $t \in \mathbb {R}$ and a vector of variables $\textbf{x} = (x_1, \ldots , x_n) \in \mathbb {R}^n$, where each $x_i$ may itself depend on $t$. In this paper, we distinguish between the *partial derivative* with respect to the explicit variable $t$, denoted by $\frac{\partial }{\partial t}$, and the *total derivative* with respect to $t$, denoted by $\frac{d}{dt}$. The partial derivative $\frac{\partial F}{\partial t}$ captures the dependence of $F$ on $t$ when $\textbf{x}$ is held fixed, whereas the total derivative $\frac{dF}{dt}$ accounts for both the explicit dependence on $t$ and the implicit dependence via the functions $\textbf{x}(t)$. In particular, the total derivative is given by $\frac{dF}{dt} = \frac{\partial F}{\partial t} + \sum _{i=1}^{n} \frac{\partial F}{\partial x_i} \cdot \frac{dx_i}{dt}.$

Next, we aim to determine the conditions under which the functional

$$\begin{aligned} Y(t, N(t; N_0)) := \frac{d}{d t} \left[ M(t) - R(t, N(t; N_0)) \right] \end{aligned}$$

is well defined, as this quantity encodes the net energetic interaction between the neighborhood and the reservoir. The sign of $Y$ plays a central interpretive role: if $Y> 0$, the neighborhood injects energy into the ecosystem at a rate that exceeds the reservoir’s capacity to retain it, suggesting an accumulation phase or external forcing. If $Y< 0$, energy is being extracted or dissipated faster than it is supplied, signaling depletion or internal consumption. This formulation shifts the focus from the separate behavior of $M'(t)$ and $\frac{d}{dt}R(t,N(t;N_0))$ to their combined effect, enabling a systemic view of ecological flux and balance.

The critical case $Y = 0$ corresponds to a dynamic equilibrium in which the energy supplied by the neighborhood is exactly balanced by the change in internal storage, i.e., the energetic state of the reservoir remains steady. In the following result, we show that if the family of measures $\mu _t$ is absolutely continuous with respect to the Lebesgue measure and the associated densities satisfy appropriate regularity assumptions. Equilibrium is achieved exclusively in the trivial case.

### Theorem 3.1

*Let the functional* $\tau _i:\mathcal {S}'\rightarrow \mathbb {R}$ be given by

$$\begin{aligned} & \tau _i\bigl (t,N(t;N_0)\bigr ) =\frac{1}{t}\int _{I_{N_0}}\bar{m}_i\bigl (s,N(s;N_0)\bigr )\\ & \,k_i\bigl (s,N(s;N_0)\bigr )\, N_i\bigl (s;N_0\bigr )\,d\mu _t(s), \end{aligned}$$

where $\{ \mu _t \}_{t> 0}$ is a family of finite positive Borel measures on each interval $[0,t]\subset \mathbb {R}_+$, absolutely continuous with respect to the Lebesgue measure. For each $t>0$, let $w_t:[0,t]\rightarrow \mathbb {R}_{\ge 0}$ be *a density of *$\mu _t$, i.e., $d\mu _t(s)=w_t(s)\,ds$. Assume that the map $(t,s)\mapsto w_t(s)$ is continuously differentiable on $D=\{(t,s)\in (0,+\infty )^2:\ 0<s<t\}$ and extends continuously to the diagonal $s=t$. Moreover, for every compact interval $[a,b]\subset (0,+\infty )$, we require *the families* $\{w_t\}_{t\in [a,b]}$ and $\left\{ \frac{\partial w_t}{\partial t}\right\} _{t\in [a,b]}$ to be uniformly integrable in $s\in [0,t]$*, and that* $w_t(t)>0$ *for all* $t>0$.

The additional assumption $w_t(t)>0$ ensures that the present state contributes with nonzero weight, so the effect is not purely delayed and includes an instantaneous component.

Then the functional

$$\begin{aligned} Y(t,N(t;N_0)) = \frac{d}{d t}\left[ M(t)-R(t,N(t;N_0))\right] \end{aligned}$$

is well defined for all $(t,N(t;N_0))\in \mathcal {S}'$. Furthermore, $Y(t,N(t;N_0))\equiv 0$ if and only if $N(t;N_0)\equiv 0$.

### Proof

Under the above hypotheses on the measures $\mu _t$ and the densities $w_t(s)$, for each *i*, it follows from Proposition 2.1 that

$$\begin{aligned}&\tau _i(t,N(t; N_0))=\frac{1}{t} \int _{I_{N_0}} \bar{m}_i(s, N(s; N_0))\\&\, k_i(s, N(s; N_0))\, N_i(s; N_0)\, d\mu _t(s) \\ =&\frac{1}{t} \int _0^t\bar{m}_i(s, N(s; N_0))\\&\, k_i(s, N(s; N_0))\, N_i(s; N_0)\, w_t(s)\, ds \end{aligned}$$

is well defined, differentiable with respect to $t$ for all $t \in (0,+\infty )$, and its derivative is given by

$$\begin{aligned} & \frac{d}{d t} \tau _i(t,N(t; N_0))= - \frac{1}{t^2} \int _0^t f_i(s)\, w_t(s)\, ds \\ & + \frac{1}{t} \left[ f_i(t)\, w_t(t) + \int _0^t f_i(s)\, \frac{\partial w_t}{\partial t}(s)\, ds \right] . \end{aligned}$$

where $$f_i(\cdot ,N(\cdot ,N_0))=\bar{m}_i(\cdot , N(\cdot ; N_0))\, k_i(\cdot , N(\cdot ; N_0))\, N_i(\cdot ; N_0)$$. Therefore,

$$\begin{aligned} & Y(t,N(t;N_0)) = \frac{d}{d t}\left( M(t) - R(t,N(t;N_0))\right) \\ & = \displaystyle \sum _{i=1}^n \frac{d}{d t} \tau _i(t,N(t;N_0))\end{aligned}$$

is well defined for all $t \in (0,+\infty )$ and $1 \le i \le n$. Now, suppose that $Y(t,N(t;N_0))= 0$ for some $N_0$* and every* $t\in I_{N_0}$. Then

$$\begin{aligned} 0&=\sum _{i=1}^{n}\frac{d}{d t}\tau _i(t,N(t;N_0)) \\&= -\frac{1}{t^2}\int _{0}^{t} \left( \sum _{i=1}^{n} f_i(s,N(s;N_0))\right) w_t(s)ds\\&+ \frac{1}{t}\sum _{i=1}^{n}f_i(t,N(t;N_0))w_t(t) \\ &+\frac{1}{t}\int _{0}^{t} \left( \sum _{i=1}^{n} f_i(s,N(s;N_0))\right) \frac{\partial w_t}{\partial t}(s)ds. \end{aligned}$$

This implies

3.2 $$\begin{aligned}&\sum _{i=1}^{n}f_i(t,N(t;N_0))w_t(t) = \int _{0}^{t} \nonumber \\&\left( \sum _{i=1}^{n} f_i(s,N(s;N_0)\right) \left( \frac{w_t(s)}{t}-\frac{\partial w_t}{\partial t}(s)\right) ds. \end{aligned}$$

*Since* $w_t(t)>0$ it follows from (3.2) that

$$\begin{aligned}&\sum _{i=1}^{n}f_i(t,N(t;N_0)) = \int _{0}^{t} \left( \sum _{i=1}^{n} f_i(s,N(s;N_0)\right) \\&\frac{\left( \frac{w_t(s)}{t}-\frac{\partial w_t}{\partial t}(s)\right) }{w_t(t)}ds. \end{aligned}$$

Consider the *Volterra Integral Equation* given by

3.3 $$\begin{aligned} \varphi (t)= \int _{0}^{t} \varphi (s)K(t,s)ds \end{aligned}$$

where $K(t,s) = \left( \frac{\frac{1}{t}w_t(s)-\frac{\partial w_t}{\partial t}(s)}{w_t(t)}\right)$. The kernel *K* is continuous on $(0,+\infty ) \times (0,+\infty )$. It then follows from (Brunner 2016, Theorem 1.2.7, pp.10) that Eq. (3.3) admits the unique trivial solution $\varphi \equiv 0$. Therefore $\sum _{i=1}^{n}f_i(s,N(s;N_0))= 0$ and, as above, *N* is trivial. $\square$

The Borel family of measures ${\mu _t}$ used in the definition of $\tau _i$ models the influence of the history of interactions on the current ecological impact of a species. Absolutely continuous measures (such as the Gaussian of Remark 3.2) assign decreasing weights to past states, simulating a memory effect where recent history has a greater influence on the present.

Note that the specific form of the GLV system is not explicitly used in this demonstration. The definition of $\tau _i$ and the derivation of *Y* depend only on the regularity of the families of measures $\{\mu _t\}$ and the functions $\bar{m}_i$, $k_i$, and $N_i(\cdot ;N_0)$. Therefore, the result regarding the well-defined *Y* and the equivalence $Y=0 \Leftrightarrow N=0$ is valid for any dynamic model that produces continuous trajectories *N*(*t*) and is not exclusive to the GLV.

### Remark 3.2

A typical example of a measure satisfying the assumptions of Theorem 3.1 is given by a Gaussian-type functional, whose density takes the form $w_t(s) = e^{-a(s - t)^2}$, with $a> 0$.

Since Theorem 3.1 established that, under absolutely continuous measures, the system reaches energetic equilibrium only in the trivial case, we are naturally led to consider alternative modeling strategies.

## Memory-free model

If the ecosystem is such that species retain virtually no memory of past interactions, the size of the species may be treated as independent of its historical context. We will model this by incorporating the Dirac delta distribution into the definition of the trophic functional.

### Proposition 4.1

Let $\tau _i:\mathcal {S}'\rightarrow \mathbb {R}$ be given by

$$\begin{aligned} & \tau _i\bigl (t,N(t;N_0)\bigr ) =\frac{1}{t}\int _{I_t}\bar{m}_i\bigl (s,N(s;N_0)\bigr )\\ & \,k_i\bigl (s,N(s;N_0)\bigr )\, N_i\bigl (s;N_0\bigr )\,d\mu _t, \end{aligned}$$

where $\mu _t = t\,\delta (t-s)$ and $\delta$ represents the Dirac delta at $0$.

Then the functional $Y:\mathcal {S}'\rightarrow \mathbb {R}$ given by

$$\begin{aligned} Y\bigl (t,N(t;N_0)\bigr ) =\frac{d}{dt}\bigl [M(t)-R(t,N(t;N_0))\bigr ], \end{aligned}$$

is well defined, where $M : [0, +\infty ) \rightarrow \mathbb {R}_{>0}$ is a prescribed function of class $\mathcal {C}^1\left( [0, +\infty )\right)$, which assigns to each time $t$ the total amount of biomass available in the ecosystem, and where $R(t, N(t; N_0)):= M(t) - \sum _{i=1}^n \tau _i(t, N(t; N_0))$.

### Proof

It follows from the basic properties of the Dirac delta that

$$\begin{aligned} \tau _i (t,N(t;N_0))&= \frac{1}{t}\int _{I_t}\bar{m}_i\bigl (s,N(s;N_0)\bigr )\\&\,k_i\bigl (s,N(s;N_0)\bigr )\, N_i\bigl (s;N_0\bigr )\,d\mu _t\\ &=\bar{m}_i (t,N(t;N_0)) N_i (t;N_0) k_i (t,N(t;N_0)). \end{aligned}$$

Since the functions $\bar{m}_i$ and $k_i$ are smooth for each $i \in \{1, \ldots , n\}$, and $N(t)$ is a solution of the system under consideration, the total derivative with respect to $t$ of each functional $\tau _i(t, N(t))$ is well defined. Consequently, the functional $R(t, N(t)) := M(t) - \sum _{i=1}^n \tau _i(t, N(t))$ is well defined, and therefore the functional $Y(t, N(t))$ is also well defined. $\square$

The Dirac distribution $(\delta (t-s))$ represents a memoryless regime, where only the instantaneous time determines $\tau _i$. This distinction is ecologically relevant: systems with memory may represent demographic inertia or resource inheritance, while systems without memory require instantaneous responses to current conditions. t

### On the harmonic property of effective trophic capacity

In the context of trophic dynamics, we define the quantity

$$\begin{aligned} p_i(t,N(t;N_0)) = \bar{m_i}(t,N(t;N_0)) \cdot k_i(t,N(t;N_0)) \end{aligned}$$

as a composite measure of species $i$’s contribution to the ecosystem. Here, $\bar{m}_i$ denotes, for each species *i*, the mean individual biomass, while $k_i$ represents its relative trophic strength. This product can be interpreted as an *effective trophic potential per individual*, capturing both the metabolic weight and the ecological influence of the species.

Inspired by principles from theoretical ecology and spatial biomathematics, we investigate the ecological and dynamical implications of assuming that $p_i$ is a *harmonic function* over a suitable ecological domain. For the sake of completeness, we recall below the standard definition of harmonic functions, as commonly found in the classical literature.

#### Definition 4.1

*(Harmonic Function)* Let $\Omega \subset \mathbb {R}^n$ be an open set. A function $u : \Omega \;\longrightarrow \; \mathbb {R}$ is said to be *harmonic* in $\Omega$ if

(i) $u\in C^2(\Omega )$, i.e., all second partial derivatives of $u$ exist and are continuous in $\Omega$;
(ii) its Laplacian vanishes at every point of $\Omega$:
  $$\begin{aligned} \Delta u(x) \;=\; \sum _{j=1}^n \frac{\partial ^2 u}{\partial x_j^2}(x) \;=\;0, \quad \forall \,x\in \Omega . \end{aligned}$$

Ecologically, harmonicity imposes the absence of internal sources or sinks in the effective trophic potential. That is, *the species operates in a state of local equilibrium*, where its contribution is smoothly integrated into the trophic network without generating abrupt concentrations or voids of trophic pressure. From a functional perspective, this reflects a *diffuse, stable ecological integration*: species $i$ neither dominates nor disappears abruptly in localized ecological contexts.

#### Theorem 4.1

Consider the Generalized Lotka–Volterra (GLV) system

4.1 $$\begin{aligned} {\left\{ \begin{array}{ll} \displaystyle {\frac{dN_i}{dt} = N_i \left( r_i + \sum _{j=1}^n a_{ij} N_j \right) , \quad i = 1, \dots , n,} \\ N(0) = N_0 \in \mathbb {R}^n_{> 0}, \end{array}\right. } \end{aligned}$$

as in (2.1). Let $\mathcal {A}\subset \mathcal {S}'$ be a subset with the property that, for each $i\in \{1,\dots ,n\}$

$$\begin{aligned} & \bar{m}_i\bigl (t,N(t;N_0)\bigr )\cdot k_i\bigl (t,N(t;N_0)\bigr ) \\ & \quad =\exp \!\Bigl (-r_i\,t \;-\;\sum _{j=1}^n a_{ij} \displaystyle \int _{0}^{t}N_j(s;N_0)\,\textrm{d}s\Bigr ) \end{aligned}$$

for all $\bigl (t,N(t;N_0)\bigr )\in \mathcal {A}$. Then the functional $Y:\mathcal {S}'\rightarrow \mathbb {R}$ given by

$$\begin{aligned} Y\bigl (t,N(t;N_0)\bigr ) =\frac{d}{dt}\bigl [M(t)-R(t,N(t;N_0))\bigr ], \end{aligned}$$

vanishes identically on $\mathcal {A}$; furthermore, if we assume that for each $i \in \{1,\dots ,n\}$, the function $p_i:= \bar{m}_i \cdot k_i$ is harmonic on $\mathcal {A} \subset \mathcal {S}'$, then

$$\begin{aligned} \sum _{i=1}^n\left[ r_i^2 + \sum _{j=1}^n a_{ij}\bigl (2r_i - r_j\bigr )N_j + \sum _{j=1}^n\sum _{k=1}^n a_{ij}\bigl (a_{ik}-a_{jk}\bigr )\,N_jN_k\right] =0. \end{aligned}$$

#### Proof

Since *N* is a solution of system (2.1), the total derivative with respect to *t* gives

$$\begin{aligned} \frac{d}{d t} \tau _i&= \frac{d}{dt}\left[ \bar{m_i}k_i\right] N_i + \bar{m_i}k_i\frac{dN_i}{dt}\\ &=\frac{d}{dt}\left[ \bar{m_i}k_i\right] N_i+\bar{m_i}k_iN_i\left( r_i+\sum _{j=1}^na_{ij}N_j\right) \\&=N_i\left( \frac{d}{dt}\left[ \bar{m_i}k_i\right] +\bar{m_i}k_i\left( r_i+\sum _{j=1}^na_{ij}N_j\right) \right) . \end{aligned}$$

Denote $p_i:=\bar{m}_i (t,N(t;N_0)) k_i (t,N(t;N_0))$ and suppose that

$$\begin{aligned}p_i= \exp \left( -r_it-\sum _{j=1}^n a_{ij}\int _{0}^{t} N_j(s;N_0)ds\right) \end{aligned}$$

for each $i\in \{1,\dots ,n\}$ and every $\bigl (t,N(t;N_0)\bigr )\in \mathcal {A}\subset \mathcal {S}'$. Then

$$\begin{aligned} \frac{d}{dt}\left[ p_i\right]&= \frac{d}{dt}\left[ \exp \left( -r_it-\sum _{j=1}^n a_{ij}\int _{0}^{t} N_j(s;N_0)ds\right) \right] \\&= \left( -r_i-\sum _{j=1}^na_{ij}N_j\right) \exp \left( -r_it-\sum _{j=1}^n a_{ij}\int _{0}^{t} N_j(s;N_0)ds\right) \\&=-\bar{m_i}k_i\left( r_i+\sum _{j=1}^na_{ij}N_j\right) . \end{aligned}$$

Therefore, for this particular choice of $\bar{m_i}$ and $k_i$ we have

$$\begin{aligned} Y&=\frac{d}{dt}\bigl [M-R\bigr ] =\frac{d}{dt}\left[ \sum _{i=1}^{n}\tau _i\right] \\&=\sum _{i=1}^{n} N_i\left( \frac{d}{dt}\left[ \bar{m_i}k_i\right] +\bar{m_i}k_i\left( r_i+\sum _{j=1}^na_{ij}N_j\right) \right) \\&=0. \end{aligned}$$

Note that, to compute the Laplacian $\Delta p_i$, we consider the tuple $(t,N_1,\dots ,N_n)$ as a set of independent variables. Since each $N_j$ is continuous in *t* and $p_i$ depends on $N_j$ only through the integrals, it follows that

$$\begin{aligned} \frac{\partial p_i}{\partial N_k}=0, \quad \frac{\partial ^2 p_i}{\partial N_k^2}=0, \quad k=1,\dots ,n. \end{aligned}$$

Hence,

$$\begin{aligned} \Delta p_i =\frac{\partial ^2 p_i}{\partial t^2} +\sum _{k=1}^n\frac{\partial ^2 p_i}{\partial N_k^2} =\frac{\partial ^2 p_i}{\partial t^2}. \end{aligned}$$

Then $$\frac{\partial p_i}{\partial t}= \bigl (-r_i-\sum _{j=1}^n a_{ij}N_j(t)\bigr )\,p_i$$ and

4.2 $$\begin{aligned} \Delta p_i = \Bigl [\bigl (-r_i-\sum _{j=1}^n a_{ij}N_j(t)\bigr )^2 \;-\;\sum _{j=1}^n a_{ij}\dot{N}_j(t)\Bigr ]\,p_i. \end{aligned}$$

Since $p_i>0$ and $p_i$ is assumed to be harmonic, it follows from (4.2) that

$$\begin{aligned} \bigl (r_i+\sum _{j=1}^n a_{ij}N_j\bigr )^2 \;-\;\sum _{j=1}^n a_{ij}\dot{N}_j =0. \end{aligned}$$

Given that *N* satisfies the GLV system (4.1), we deduce that

$$\begin{aligned} & r_i^2 + 2r_i \sum _{j=1}^n a_{ij}N_j + \sum _{j=1}^n\sum _{k=1}^n a_{ij}a_{ik}N_jN_k \\ & \quad - \sum _{j=1}^n a_{ij}N_j\left( r_j+\sum _{l=1}^na_{jl}N_l\right) =0, \end{aligned}$$

and hence the identity above expands to

$$\begin{aligned} & \sum _{i=1}^n\bigg [r_i^2 + \sum _{j=1}^n a_{ij}\bigl (2r_i - r_j\bigr )N_j\\ & \quad + \sum _{j=1}^n\sum _{k=1}^n a_{ij}\bigl (a_{ik}-a_{jk}\bigr )\,N_jN_k\bigg ]=0. \end{aligned}$$

$$\square$$

#### Remark 4.1

In contrast to the memory-dependent ecosystem treated in Theorems 3.1, 4.1 shows that, in the memory-free ecosystems, there exist nontrivial regions of the phase space in which the competing rates *M* and *R* are at equilibrium.

## Bidimensional case

Let us now exemplify our general framework by describing the phase space dynamics of an ecosystem containing two interacting species (prey and predator) where resources are shared with the harmonic property of effective trophic capacity, and there is no memory effect.

Consider the Lotka–Volterra system

5.1 $$\begin{aligned} {\left\{ \begin{array}{ll} \displaystyle \frac{dN_1}{dt} = N_1 \left( r_1 + a_{11} N_1 + a_{12} N_2 \right) , \\ \displaystyle \frac{dN_2}{dt} = N_2 \left( r_2 + a_{21} N_1 + a_{22} N_2 \right) , \\ N(0) = N_0 \in \mathbb {R}^2_{> 0}, \end{array}\right. } \end{aligned}$$

where $\mathbb {R}^2_{> 0} = \{ x \in \mathbb {R}^2: x_1> 0,\; x_2> 0 \}.$ It follows immediately from Theorem 4.1 that, for $n=2$, we have

$$\begin{aligned}&r_1^2 + r_2^2 + N_1\bigl [a_{11}r_1 + a_{21}(2r_2 - r_1)\bigr ] \\&\quad + N_2\bigl [a_{12}(2r_1 - r_2) + a_{22}r_2\bigr ] \\ &\quad + N_1^2\bigl [a_{21}(a_{21} - a_{11})\bigr ] \\&\quad + N_1N_2\bigl [a_{12}(a_{11} - a_{21}) + a_{21}(a_{22} - a_{12})\bigr ] \\ &\quad + N_2^2\bigl [a_{12}(a_{12} - a_{22})\bigr ] =0. \end{aligned}$$

In a simplified form, we can represent the system dynamics by a general equation:

5.2 $$\begin{aligned} x\left[ 1 +c_1 x + c_2 y \right] +c_3 y\left[ 1+c_4 y +c_5 x\right] = c_6 \end{aligned}$$

where *x* and *y* represent the populations $N_1$ and $N_2$ in a normalized phase space, $c_1,c_2,c_3,c_4,c_5$, and $c_6$ and are constants.

The coefficients $c_1, \dots , c_6$ in Eq. (5.2) are derived directly from the application of Theorem 4.1 to the case $n=2$ and are related to the original parameters of the GLV (Eq. 5.1) as follows:

$$\begin{aligned}\begin{aligned} c_1&= \frac{a_{21}(a_{21} - a_{11})}{\mathcal {K}}, \quad c_2 = \frac{a_{12}(a_{11} - a_{21}) + a_{21}(a_{22} - a_{12})}{\mathcal {K}}, \\ c_3&= \frac{a_{12}(a_{12} - a_{22})}{\mathcal {K}}, \quad c_4 = \frac{a_{12}(2r_1 - r_2) + a_{22}r_2}{\mathcal {K}}, \\ c_5&= \frac{a_{11}r_1 + a_{21}(2r_2 - r_1)}{\mathcal {K}}, \quad c_6 = -\frac{r_1^2 + r_2^2}{\mathcal {K}}, \end{aligned}\end{aligned}$$

where $\mathcal {K}$ is a positive normalization factor that emerges from the algebraic simplification process. This explicit translation reveals that the harmonicity condition, $\Delta p_i = 0$, imposes a global quadratic constraint between the population densities $N_1$ and $N_2$.

While classical analysis of dynamical systems focuses on equilibrium points, Jacobian matrix eigenvalues, and the existence of limit cycles, our harmonicity-derived framework identifies specific algebraic curves in phase space (given by Eq. (5.2)) where the net energy flow *Y* vanishes. This allows us to characterize entire regions of sustainable coexistence that depend in a non-trivial way on both intrinsic rates $r_i$ and the interaction structure $a_{ij}$. Thus, the present approach complements traditional dynamical analysis by providing a geometric–algebraic criterion for stability based on the energy balance between the biotic and abiotic components of the ecosystem.

The constants contain the interaction effects and population growth/decay rates, allowing us to explore general behaviors. For specific values of $c_i$, closed, elliptical trajectories emerge in phase space, known as Closed Orbit Trajectories or Periodic Orbits (Almaraz et al. 2024). These stable, oscillatory paths represent the fundamental cyclical nature of predator/prey interactions, where populations perpetually fluctuate around an equilibrium point while maintaining a constant phase relationship, with predator peaks consistently lagging behind prey peaks. The closed nature of these orbits indicates that the system periodically returns to specific population configurations, creating sustainable predator/prey cycles that could theoretically continue indefinitely in this idealized model.

In other parameter ranges, the model produces hyperbolic trajectories (open curves), suggesting divergence in population values, which could indicate instability or unsustainable interaction where one species potentially grows uncontrollably or collapses, leading to system imbalance. The distinction between these closed and open trajectories is crucial for understanding population dynamics, with the closed orbits representing balanced ecosystems and the open curves warning of potential ecological collapse. These behaviors are further examined in the unifying framework by Van Meerbeek and Jucker (2021).

The elliptical closed orbits specifically demonstrate how predator and prey populations are intrinsically linked through their interaction coefficients, with the shape and size of each orbit determined by initial population sizes and the strength of species interactions. These periodic solutions highlight the delicate balance required for ecological stability and serve as a mathematical representation of the observed population cycles seen in many natural systems.

To illustrate the stability and viability of solutions in a predator/prey model, we analyze the cyclical dynamics of wolf ($N_2$) and rabbit ($N_1$) populations, as documented in Zill et al. (1997), Boyce et al. (2017), Stewart et al. (2021). Using the phase plane method, a valuable tool for mapping solution trajectories, we examine whether the nonlinear system’s coexistence equilibrium point functions as a center, as described in Eq. 5.2. The first data analyzed in what follows come from the sole patterns arising from the harmonicity equation coined in Eq. 5.2. The trajectories form open and closed curves around the equilibrium point (1000, 80) for all tested initial conditions (e.g., (0, 0)). Each initial condition yields a distinct periodic curve, confirming that the equilibrium point is indeed a center for the nonlinear system.

**Fig. 1:**
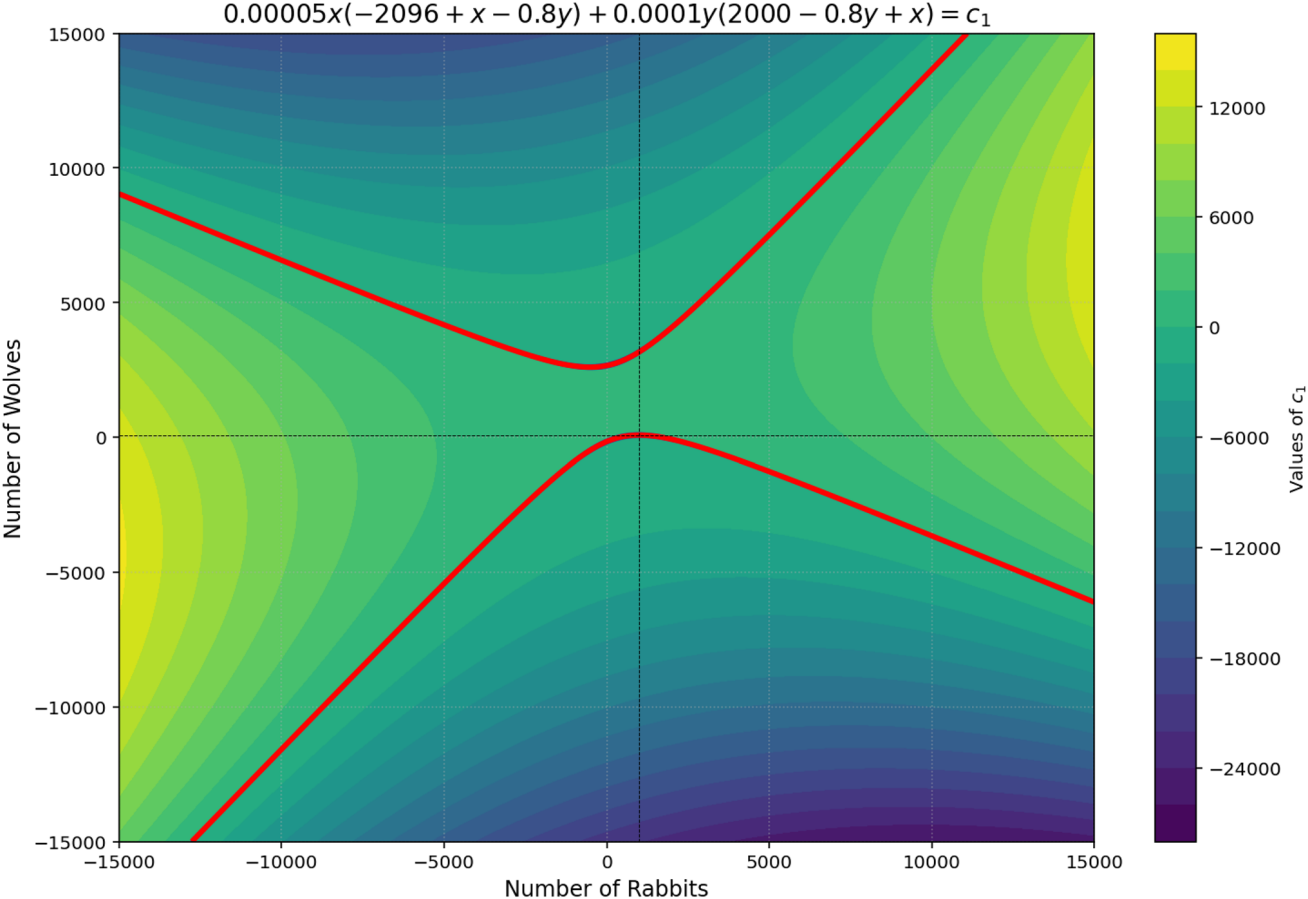
Relations between the populations $N_1$ and $N_2$ are unstable for a positive sign in the term $N_1^2$; in this case, the prey must compete.

Figure 1 shows the system’s instability when the term $N_1^2$ has a positive sign, indicating that prey species inherently compete, driving population growth to unsustainable levels (hyperbolic trajectory). Stability is only achieved when both $c_1$ and $c_4$ are negative, suggesting that intra-species interactions must be competitive (density-limited) for equilibrium to emerge.

Furthermore, a stable interaction between the predator and the prey requires $c_2<0$ and $c_5>0$, meaning the predator benefits more from the interaction, leading to controlled growth of the predator population and balanced ecological exchange.

**Fig. 2:**
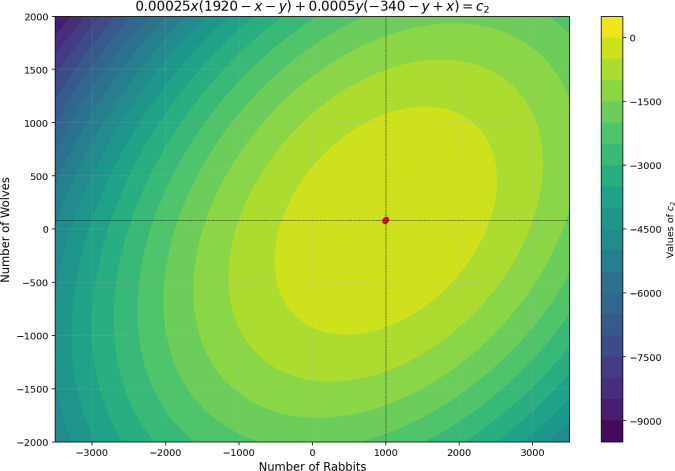
Relation between prey and predator populations assumes negative mathematical values, indicating a lack of valid values. The relation shows some invalid values for the predator population. The lack of valid values may come from inadequate values for the prey/predator mean masses or the balance between mass and information. Other relations for a stable configuration of the system.

Figure 2 shows stable closed-loop behavior, where populations oscillate within limits, maintaining dynamic equilibrium (elliptical trajectory). In this scenario, the system reaches a stable balance, maintaining predator/prey interactions without unbounded growth or decline. In addition, we observe negative or otherwise invalid values for population sizes, indicating scenarios where the configuration of mean masses, mass information balance, or parameter signs is not conducive to realistic positive populations.

The term $\frac{d}{dt}[M-R]$ can alter stability, with stability being more common when this term is positive. This suggests that the organized inflow of information (representing a structured ecological input) tends to stabilize the system (see Fig. 2).

Since *M* and *R* vary over time, the system’s stability is subject to changes in entropy and mass information flux. Consequently, the system could shift between stable and unstable states, depending on the time-evolving balance of these parameters. Figure 2 illustrates that with appropriate parameter values, the model can reach various stable configurations, but these conditions are sensitive to shifts in the balance of competitive dynamics, organized information influx, and ecological mass.

**Fig. 3:**
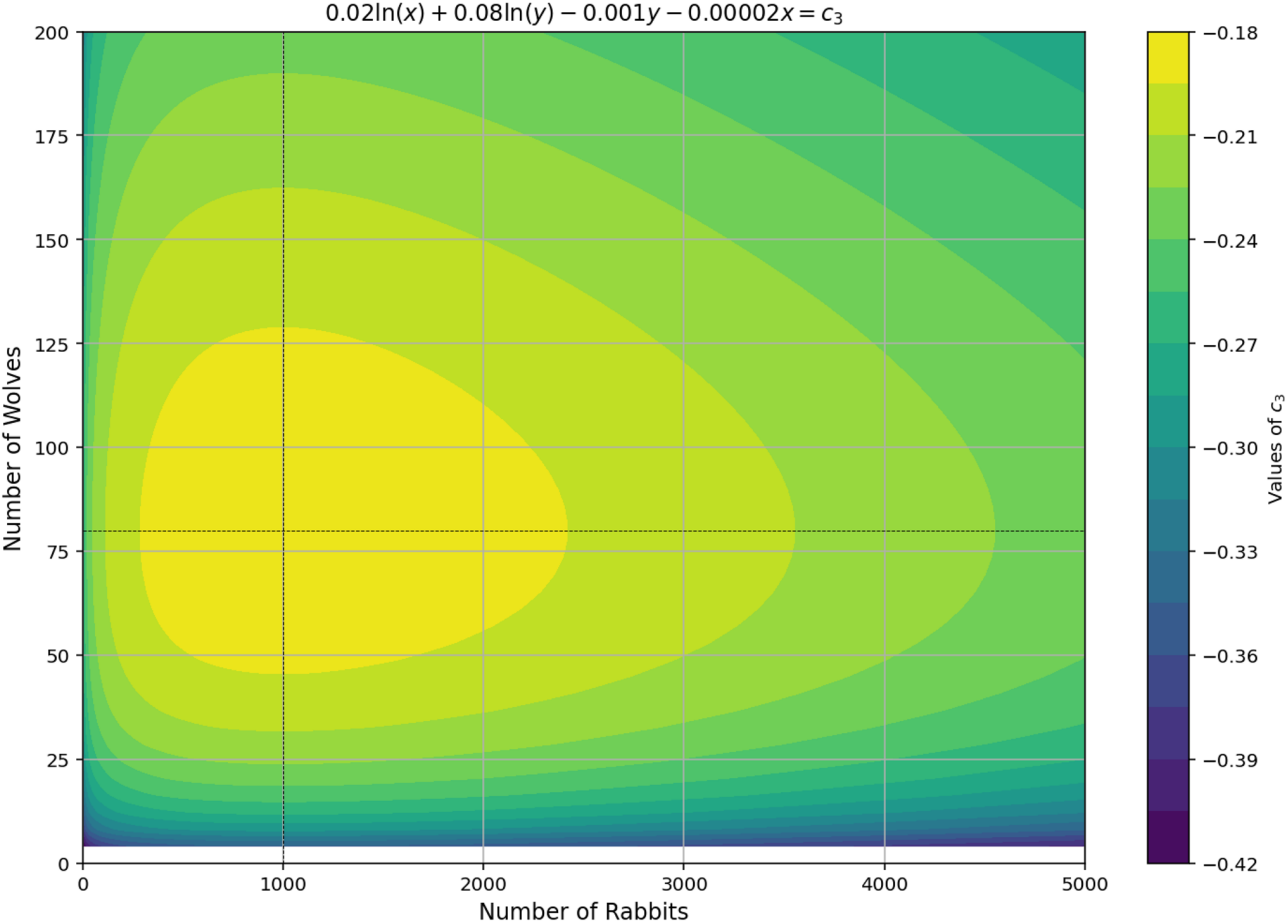
Relations between the populations $N_1$ and $N_2$ are stable and periodic curves around the equilibrium point.

This is to be compared to the resulting solutions coming from the GLV equations. Figure 3 depicts the phase portrait of the cyclical dynamics between wolf and rabbit populations for the classical logarithm solution to the pure GLV system of derivatives, where trajectories form closed, periodic curves around the equilibrium point. Notably, the rabbit population fluctuates before the wolf population, as the initial scarcity of predators allows the rabbit population to grow. With more prey available, the wolf population also increases, intensifying predation and reducing the number of rabbits. As food becomes scarce, the wolf population declines, eventually returning the system to its original state. The average populations over a full cycle are 80 wolves and 1000 rabbits, matching the equilibrium values.

As may be observed in studies such as Holling (1973), the patterns here observed align with those typically obtained in the literature to date. However, one should stress that the shapes arising from the derivatives attained to the GLV system and the ones coming from the harmonicity condition must be, at some point, confronted with each other and calculated simultaneously. That is, in some sense, the same as demanding the dynamical conversion between different patterns as the limit circles evolve, all driven by the energy constraint on the system. Such a restrictive imposition of dynamical evolution resembles a more fundamental Hamiltonian evolution construction from the outset. It must explore a far richer number of patterns achievable for the different regimes of income/outcome of energy into the system, which must be explored in detail in future works.

## Energy and mass flow

The continuity equation, $Y =M' - R'$, allows a deductive work of understanding the driving dynamics exerted by the source of external energy. An analysis of mass and energy exchanges between the neighborhood, the ecosystem, and even the trophic and molecular relationships between the living organisms and the organic reservoir may be examined through an analysis of the *Y* function and the other quantities. A positive *Y* indicates an overall increase in the combined prey/predator populations, provided the force equation $Y=\sum _i \tau _i'$.

**Fig. 4:**
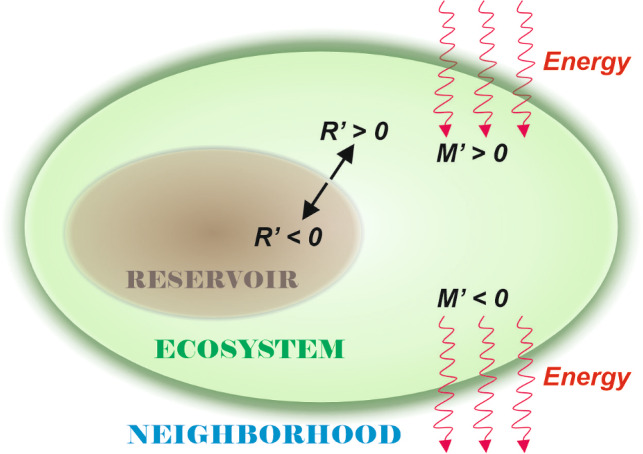
Flux of energy and matter in the system: The neighborhood may introduce energy in the ecosystem ($M^{\prime }>0$) either withdraw energy ($M^{\prime } <0$). Also, the resources stocked in the reservoir may be added, and the reservoir will expand ($R^{\prime }>0$); otherwise, the reservoir may lose resources and contract ($R^{\prime }<0$). Here, the arrows for the reservoir represent its growth or shrinkage, instead of income or outcome of mass energy.

Figure 4 sketches the energy packs exchanged between the main actors here displayed: the ecosystem itself, containing the web, and the reservoir of organic matter and, beyond, an external neighborhood. The exchanges there represented are generated via several dynamical mechanisms described *below*.

Next, we show the summarized schemes assumed by the energy flowing between the ecosystem and the neighborhood elements: the actors playing some role in the game are the food chain, the organic reservoir, and the neighborhood (Table 1).

The positive energy input (endothermic regime $Y>0$) is utilized to simultaneously increase the reservoir R and the populations C (Scenario (**a**) in Table 2); the energy input from the neighborhood N, along with resources extracted from the diminishing reservoir R, drives the population increase (Scenario (**b**) in Table 2); depleting the stock of reservoir releases energy to the environmental neighborhood (exothermic regime), contributing additional resources that enhance population growth (Scenario (c) in Table 2).

**Table 1:**
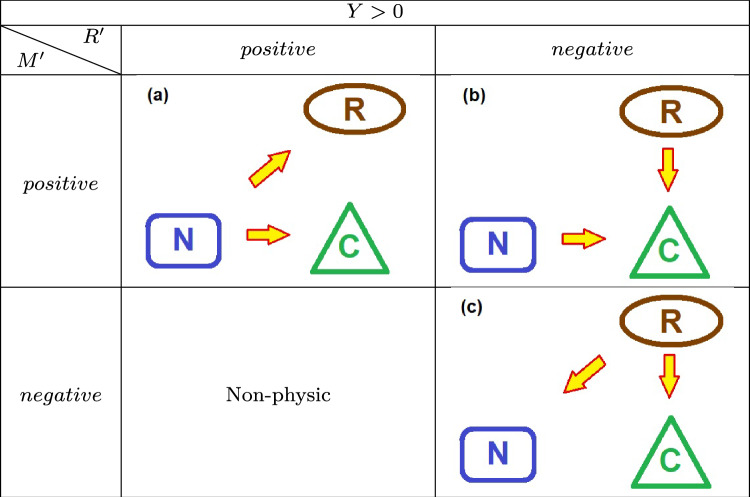
Scenarios for positive *Y* values.

**Table 2:**
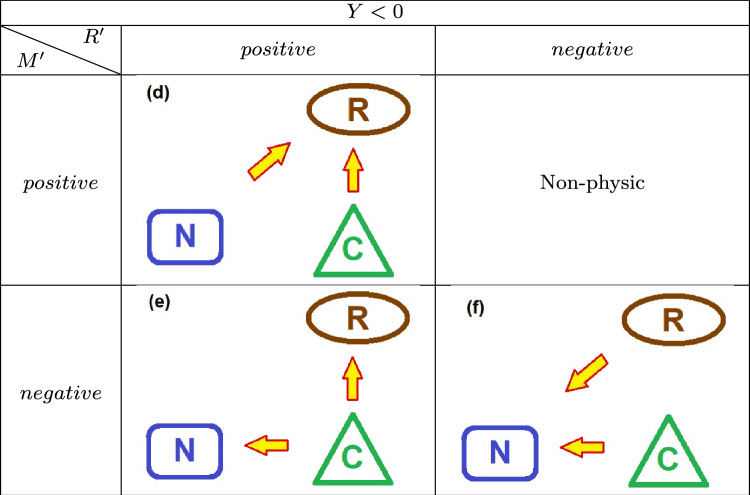
Scenarios for negative *Y* values.

**Table 3:**
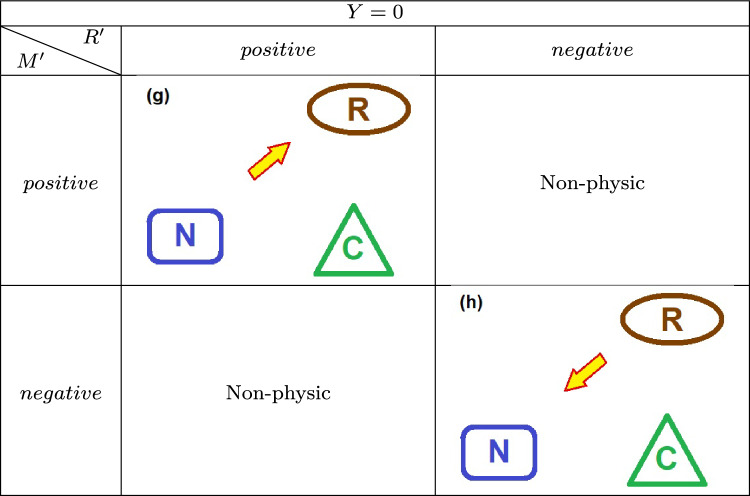
Scenarios for vanishing *Y* values.

A negative *Y* reflects a decrease in the total prey/predator population C. The possible scenarios are described in Table 3. Environmental energy N is absorbed; meanwhile, populations decline to enhance the reservoir’s growth (Scenario (**d**)); the population reduction contributes to an increase in the reservoir biomass and releases energy to the environment (exothermic regime)(Scenario (**e**)); and both populations decrease, with the reservoir being depleted to release energy to the environment, further accelerating the population decline (Scenario (**f**)).

The populations remain constant for $Y = 0$, implying a steady state. The scenarios are presented in Table 3, and in this case one has . For positive energy input (endothermic condition) and a positive rate of change in the reservoir ($R^{\prime }>0$), all incoming energy is directed toward enhancing the reservoir (Scenario (**g**)) and for negative energy input (exothermic condition) and a decreasing $R^{\prime }$, the reservoir is depleted, with the corresponding energy loss being transferred to the environment (Scenario **(h)**).

## Discussions and perspectives

The GLV model, enriched by finite resource constraints and the explicit $M-R$ balance, generates rich mathematical patterns in phase portraits that expand upon classical results from predator/prey system and food web analysis. In particular, the new pattern exhibited refers to the geometric transition between closed elliptical orbits (stable cycles) and open hyperbolic trajectories (divergence), as illustrated in Figs. 1 and 3. This transition is governed not only by the classical interaction parameters $a_{ij}$, but also by the sign and magnitude of the term $M'-R'$, which represents the net and organized flow of information and mass between the abiotic reservoir and the trophic network.

Unlike standard linear stability analysis, which focuses on the neighborhood of equilibrium points, our framework, derived from the harmonicity of the effective trophic potential ($\Delta p_i = 0$) and reservoir dynamics *R*, reveals how the global structure of the phase space is shaped. The harmonicity condition, when combined with resource constraint, can generate deformed cycles, assuming shapes that deviate from the perfect ellipse (e.g., configuring an approximate rounded "triangle" shape) in certain parameter regimes. These deformations deserve further investigation, as they may correspond to ecological regimes where intraspecific competitive pressure and resource use efficiency create pronounced asymmetries in population fluctuations.

The ability of our unified framework to explicitly link the geometry of trajectories in phase space with the fundamental thermodynamic principles (mass and energy balance) and with a global mathematical property (harmonicity) goes beyond conventional local analysis.

As to the harmonicity condition, one can still focus on the geometrical meaning of the formulation and the consequences for the mechanical treatment. The p-number $\tau _i$ here involved with a dynamical force equation will not fulfill the Laplace condition $\Delta \tau _i =0$ since the external forces must work as a source of dynamics for the Poisson equation. Nevertheless, the "individual" p-numbers $p_i = \bar{m}_i k_i$ have been treated here harmonically across the ecological phase space, so that $\Delta p_i =0$ corresponds to a valuable equivalence for some specific regime. This may be interpreted as a statement that the system dynamics manifests itself as an exclusively collective phenomenon. Such an assumption, rooted in the absence of internal trophic sources or sinks, leads to tractable analytical conditions and reveals intrinsic stability properties of the underlying system.

The constraint brought by the bounded resources condition, along with the consideration of biomass formation from inorganic matter, and biological mass distribution between species and the organic biomass reservoir have furnished a novelty for the framework. By recognizing finite resources, we highlight that competition within ecosystems constrains species growth and drives a competitive equilibrium based on biomass availability.

The new pattern exhibited by the phase portrait must be further investigated so that we can clarify in detail, for instance, the transition between the ellipsoidal shape and the deformed ’triangle’ shape in the limit cycles. In this sense, we must also get a deeper analysis upon the stable conditions pertaining to the coefficients in the GLV. In order to do that, we will produce a broad investigation upon the first principles involved with the formation of the coefficients: those will be yielded from the informational content of each individual species as a consumer and maker of energy and entropy. The entropy considerations should account for real-world informational shifts due to human and environmental impacts, positioning information flow as a driver of ecosystem stability or instability. In this sense, a sensitive model that can deal with stability through variable coefficient dynamics, diverging from previous studies by allowing open-system interactions that include immigration, which may sustain stability under specific conditions (Naji and Balasim 2007), may be a worthwhile effort.

On the other hand, we have found an interesting result recalling the memory or memory-free character of the systems. This may be further investigated, as long as the absence of a stable equilibrium condition with $Y=0$ is a remarkable fact.

Another interesting feature may be addressed in future work, namely the issue of sexual gender, as it may provide deeper insights into the nature of intra-species interactions. Specifically, when individuals interact with one another, two possible outcomes arise: *a)* Same gender: competition for resources, *b)* Different genders: cooperation through mating.

Finally, a proper question about the classical treatment of the bi-dimensional case: to what extension does the approximation used to consider a simple system composed solely of a prey and a predator as a valid framework to model reality? Such a system, isolated from the world, perfect and independent of external biomass and energy, does not even exist. So, the point here is: under which conditions do the results obtained from this toy model correspond to the real data, and why? Approaching such a question may give rise to important questions about the reservoir in the continuity condition. The conjecture suggests that the dynamical relationship between the two species can be understood through a rescaling of the reservoir, where all other species contribute to the biomass reservoir for the two focal species. Future work may also focus on this conjecture, which forms a fundamental basis for studying the interactions of two competing species.

## Data Availability

No datasets were generated or analyzed during the current study.

## Code availability

The code generated and analyzed during the current study is available from the corresponding author upon reasonable request. Access to code may be subject to ethical considerations and institutional policies.
